# AhR Activation Leads to Attenuation of Murine Autoimmune Hepatitis: Single-Cell RNA-Seq Analysis Reveals Unique Immune Cell Phenotypes and Gene Expression Changes in the Liver

**DOI:** 10.3389/fimmu.2022.899609

**Published:** 2022-06-03

**Authors:** Alkeiver S. Cannon, Bryan Latrell Holloman, Kiesha Wilson, Kathryn Miranda, Nicholas Dopkins, Prakash Nagarkatti, Mitzi Nagarkatti

**Affiliations:** Department of Pathology, Microbiology, and Immunology, University of South Carolina School of Medicine, Columbia, SC, United States

**Keywords:** TCDD, aryl hydrocarbon receptor (AhR), liver injury, scRNA seq, inflammation, autoimmune hepatitis

## Abstract

The aryl hydrocarbon receptor (AhR) is a ubiquitously expressed ligand-activated transcription factor. While initially identified as an environmental sensor, this receptor has been shown more recently to regulate a variety of immune functions. AhR ligands vary in structure and source from environmental chemicals such as 2,3,7,8-Tetrachlorodibenzo-p-dioxin (TCDD) and indoles found in cruciferous vegetables to endogenous ligands derived from tryptophan metabolism. In the current study, we used TCDD, a high affinity AhR ligand to study the impact of AhR activation in the murine model of autoimmune hepatitis (AIH). Primarily, we used single-cell RNA-sequencing (scRNA-seq) technology to study the nature of changes occurring in the immune cells in the liver at the cellular and molecular level. We found that AhR activation attenuated concanavalin A (ConA)-induced AIH by limiting chemotaxis of pro-inflammatory immune cell subsets, promoting anti-inflammatory cytokine production, and suppressing pro-inflammatory cytokine production. scRNA-seq analysis showed some unusual events upon ConA injection such as increased presence of mature B cells, natural killer (NK) T cells, CD4+ or CD8+ T cells, Kupffer cells, memory CD8+ T cells, and activated T cells while TCDD treatment led to the reversal of most of these events. Additionally, the immune cells showed significant alterations in the gene expression profiles. Specifically, we observed downregulation of inflammation-associated genes including *Ptma*, *Hspe1*, and *CD52* in TCDD-treated AIH mice as well as alterations in the expression of migratory markers such as *CXCR2*. Together, the current study characterizes the nature of inflammatory changes occurring in the liver during AIH, and sheds light on how AhR activation during AIH attenuates liver inflammation by inducing phenotypic and genotypic changes in immune cells found in the liver.

## Introduction

Autoimmune hepatitis (AIH) is an immune-mediated liver disease characterized by circulating autoantigens, elevated immunoglobulin G (IgG) and aminotransferase levels, and interface hepatitis (1). During AIH development, a lack of self-tolerance increases the number autoreactive CD4+ and CD8+ T cells, driving excess inflammation and culminating in the sustainment of hepatitis (2). It is believed that this failure of immune tolerance to liver antigens is caused by a combination of environmental factors and genetic predispositions that trigger primarily T cell-mediated inflammation (1, 2). AIH is a major health concern in the United States as approximately 6% of all liver transplants are due to autoimmune hepatitis (3). While AIH like many autoimmune disorders is characterized by a predisposition for the female gender, recent studies show that its prevalence worldwide is increasing predominantly in males (1). Treatment regimens for AIH include immunosuppressants and corticosteroids such as azathioprine and prednisone (4), however, the disease often progresses to cirrhosis and end-stage liver disease despite treatment (5). Thus, more studies are required in hopes of understanding the mechanisms through which immune cells trigger an autoimmune response against the liver, and to develop a more specific treatment approach.

The aryl hydrocarbon receptor (AhR) is a ligand-activated, cytosolic transcription factor frequently implicated in shaping the immune response (6–8). Studies have shown that certain AhR agonists, specifically the prototypic ligand, 2,3,7,8-Tetrachlorodibenzo-p-dioxin (TCDD), are highly immunosuppressive (9) and shift the T helper 17 (Th17)/Foxp3+ T regulatory cell (Treg) balance towards a Treg dominated landscape (10–12). TCDD is an environmental pollutant that has consistently exhibited suppression of inflammation through the induction of immune cell apoptosis (12, 13), suppression of cytokines (14), and differentiation of immunosuppressive cells, such as Myeloid-Derived Suppressor Cells (MDSCs) (15) and Tregs (8, 16). Thus, AhR serves not only as an environmental sensor but also as a regulator of the immune response. In the current study, therefore, we investigate by using TCDD, the role of AhR activation on a murine model of hepatitis.

Concanavalin A (ConA) is a T cell mitogen that when injected *in vivo*, activates a large number of T cells which produce cytokines that further activate other immune cells, creating symptoms reminiscent of autoimmunity (17). ConA injection in mice is utilized to induce experimental hepatitis that mimics human AIH (18). Because of the involvement of different types of immune cells during ConA-induced hepatitis, it is important to know the relative roles played by these cells in hepatitis and how AhR activation will impact these cell types.

In the current study, therefore, we used scRNA-seq on cells infiltrating the liver during ConA-induced hepatitis to identify the nature of immunological changes occurring in the liver during the injury and identify the transcriptomic changes in each cell type. We found that in addition to the expected changes in T cell subsets caused by ConA, we noted unique changes occurring in B cells, Kupffer cells, endothelial cells, and the like. Also, AhR activation by TCDD attenuated ConA-induced hepatitis and additionally, reversed cellular and molecular changes brought about by ConA.

## Materials and Methods

### Mice and ConA Treatment

C57/Bl6 mice, 8 – 10-week-old (JAX Stock # 000664) were obtained from The Jackson Laboratory and were housed in an AAALAC-accredited, specific-pathogen-free facility at the University of South Carolina School of Medicine. ConA-induced hepatitis was carried out as described previously (15, 17, 19, 20). Mice were challenged with 12.5 mg/kg body weight of Concanavalin A (catalog number: L7647, Sigma) in phosphate-buffered saline (PBS) or Vehicle (PBS) by intravenous injection. One hour after challenge, mice were treated with 10 μg/kg TCDD or corn oil (vehicle), as described (15, 17, 19, 20). TCDD was gifted by Dr. Steve Safe (Department of Veterinary Physiology and Pharmacology, Texas A&M University, College Station, TX). At the conclusion of the study, designated as 24 hours after ConA challenge, mice were euthanized by overdose isoflurane inhalation. All experiments were performed according to protocols approved by the University of South Carolina Institutional Animal Care and Use Committee.

### Histology

Euthanized mice were perfused with 10 mL of heparinized PBS before liver tissues were removed. Liver tissues were fixed in 4% paraformaldehyde for 24 hours, placed in 70% ethanol for 24 hours, and then in 1% paraformaldehyde for 24 hours before being embedded in paraffin. Hematoxylin and eosin (H&E) staining of liver tissue sections were performed under standard protocols by the University of South Carolina Instrumentation Resource Facility. Slides were examined by light microscopy for infiltrating leukocytes and tissue injury.

### Analysis of ALT Levels

Blood was obtained from euthanized mice 24 hours after ConA injection. Serum was isolated by centrifugation and collected. The activity of liver enzyme ALT in the serum was determined by a spectrophotometric method using a commercially available assay kit (Pointe Sci. Inc., Pointe-Claire, QC, Canada), as described previously (20).

### Isolation of Liver Mononuclear Cells

Liver mononuclear cells were isolated using the liver dissociation kit provided by Miltenyi Biotec (Bergisch Gladbach, Germany). Briefly, whole liver tissue was placed in a C tube with a pre-warmed dissociation mix and placed on the gentleMACS™ dissociator for homogenization. The sample was incubated at 37°C for 30 minutes under continuous rotation at 100 rpm before being placed back on the gentleMACS™ dissociator. The cell suspension was then filtered and washed with 5 mL Dulbecco’s Modified Eagle Medium (DMEM). Centrifugation at 300xg and room temperature (25°C) was performed for 10 minutes before the supernatant was discarded. The cell pellet was then resuspended into 6 milliliters (mL) of staining buffer (PBS with 2% heat-inactivated FBS and 1 mM EDTA) and transferred to a 15 mL conical tube containing 3 mL of 100% Percoll. The cells were then resuspended in the resulting 33% Percoll solution were placed in the centrifuge at 2000 revolutions per minute (rpm) for 15 minutes at room temperature. After discarding the supernatant, red blood cell lysis was performed for 5 minutes on ice and washed with staining buffer. The resulting single-cell suspension was filtered and counted using a Bio-Rad TC20 Automated Cell Counter (Hercules, CA).

### ELISA Quantification of Secreted Cytokines

Liver mononuclear cells were isolated as described above and plated in 96-well plates at a concentration of 2 × 10^6^ cells/well. After twenty-four hours, the culture supernatants were isolated for enzyme-linked immunoassay (ELISA) quantification of cytokines such as interleukin-2 (IL-2), interleukin-10 (IL-10), and interleukin-17A (IL-17) production. Additionally, splenocytes were cultured with 5µg/mL ConA for one hour before the addition of 100nM TCDD. Twenty-four hours after culture with ConA, the culture supernatants were harvested to measure cytokines IL-10 and interleukin-22 (IL-22). Serum was isolated from blood obtained at the time of euthanization and used to perform ELISA to detect IL-6. ELISAs were performed according to BioLegend (San Diego, CA) protocol. In brief, high-affinity protein-binding plates were coated by incubating a 100 microliter (µL) suspension of capture antibody overnight at 4°C. The plates were then washed four times using a wash solution consisting of 1xPBS + 0.05% Tween80. The plates were incubated with blocking solution for 1 hour at room temperature (RT). After incubation, the plates were washed four times using wash solution and incubated for 2 hours at RTI with 100 µL of standards prepared using serial dilutions according to manufacturer’s instructions or 100 µL of supernatant collected from overnight cell cultures or the serum. Plates were again washed four times with wash solution and incubated with 100 µL of a biotinylated detection antibody solution diluted according to the manufacturer’s instruction at RT for 1 hour. After incubation with the detection antibody, plates were washed four times with wash solution and incubated with horseradish peroxidase (HRP) conjugated avidin antibody for 30 minutes at RT. After this incubation, the plates were washed five times with wash solution prior to incubation with 100µL 3,3’,5,5’-Tetramethylbenzidine (TMB) substrate for 20 minutes in the dark for color development. After color development, the reaction was stopped in all wells *via* the addition of one volume of 1N hydrosulfuric acid. The concentration of captured protein content was calculated by comparing the relative absorbance of variable samples at a wavelength of 450 nanometers to the standard curve calculated from standards of known concentration. Plates were analyzed with a PerkinElmer Victor^2^ plate reader.

### Flow Cytometry

Cell number and viability of liver mononuclear cells were quantified using a TC20 Automated Cell Counter from Bio-Rad, then washed in staining buffer for characterization *via* flow cytometry. Blocking of Fc receptors was performed by incubation with TruStain FcX (BioLegend) for 10 minutes. Next, the cells were incubated with appropriate fluorochrome-conjugated antibodies for 30 minutes on ice (CD45-APC/Cy7, clone: 30-F11; CD3ε-FITC, clone: 145-2C11; CD4-BV786, clone: GK1.5; FoxP3-BV421, clone: MF-14 from BioLegend and RORyt-PE, clone: Q31-378 from BD Pharmingen). Cells were washed with staining buffer and then analyzed on a BD FACSCelesta flow cytometer. Data were analyzed with FlowJo v10 software.

### Single Cell RNA-Sequencing (scRNA-Seq)

Liver mononuclear cells were loaded onto the Chromium Controller (10x Genomics) targeting 3000 cells per lane. The Chromium Next GEM single-cell 5’ reagent kit v2 (Dual Index) was used to process samples into single-cell RNA-seq libraries according to the manufacturer’s protocol. Libraries were sequenced with a NextSeq 550 instrument (Illumina) with a depth of 30,000 – 41,000 reads per cell. The 10x Genomics Cell Ranger pipeline (version 3.0.2) was used to generate FASTQ files, align reads to mm10 genome, and summarize read count for each gene per individual cell. Downstream analysis was completed using Loupe Browser (version 5.1) and Partek Flow. Differential expression was determined for each cluster to determine cluster biomarkers, and between the Naïve, ConA+Veh, and ConA+TCDD samples using sSeq method (21) and the Benjamini-Hochberg procedure to correct for the false discovery rate.

### Statistical Analysis

The number of mice used in each group has been depicted in the Figure. Statistical analyses were performed using GraphPad Prism Version 9.000 for Mac (GraphPad Software). Values were expressed as mean ± standard error of the mean (SEM). One-way ANOVA followed by Tukey’s posthoc test was used for multiple group analyses. *P* < 0.05 was considered statistically significant.

## Results

### Treatment With TCDD Ameliorates ConA-Induced Liver Injury

**Figure 1:**
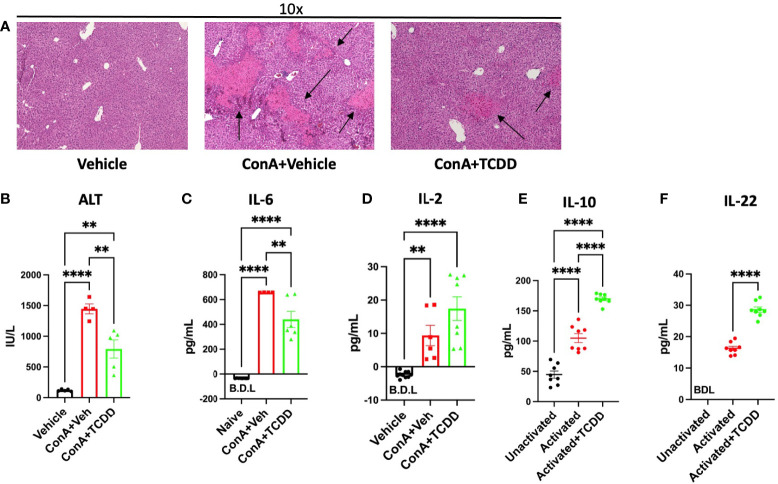
Treatment with TCDD ameliorates ConA-induced liver Injury. C57/BL6 mice were injected with ConA followed by vehicle or TCDD 1 hour later as described in Methods. After 24 hours, livers were perfused, sectioned, and stained with H&E and serum from blood collected. **(A)** Histopathological analysis shows infiltrating cells in the liver of ConA-treated mice when compared to the vehicle controls and TCDD treated group showing decreased infiltration of cells. Damaged areas are indicated by arrows **(B)** ALT levels in serum. **(C)** IL-6 levels and **(D)** IL-2 levels in the serum measured by ELISA. **(E)** Splenocytes were activated *in vitro* with ConA and exposed to TCDD, and the culture supernatants were assayed for IL-10 or **(F)** IL-22 by ELISA. Significance was determined by one-way ANOVA followed by Tukey’s posthoc test. Vertical bars represent Mean +/- SEM. **p < 0.01, ****p < 0.0001.

In order to determine how AhR activation alters liver damage in AIH, we first monitored histopathological progression of liver damage. H&E staining of liver tissue demonstrated that ConA+Vehicle treated mice display significant symptoms of liver damage, while treatment with TCDD decreases immune cell infiltration and associated damage parameters (**Figure 1A**). TCDD treatment also reduced areas of liver damage present in the ConA+Vehicle treated mice as evidenced by the healing of areas of disrupted tissue architecture (**Figure 1A**). TCDD treatment significantly alleviated ConA induced liver damage as detectable by circulating levels of ALT (**Figure 1B**).

Next, we tested the levels of IL-6 in the serum which has been shown to cause damage to the liver cells during ConA-induced hepatitis (22). IL-6, an indicator of T cell proliferation, was increased following ConA injection while TCDD treatment caused a significant decrease in its level (**Figure 1C**). IL-2 secretion was increased following both ConA injection and TCDD treatment (**Figure 1D**). To test if TCDD caused a direct effect on the T cells, we cultured splenocytes *in vitro* with 5 μg/mL ConA, and after 1 hour, TCDD was added at a dose of 100nM. Supernatants were collected after 24 hours and ELISAs for IL-10 and IL-22 were conducted. We observed a significant increase in IL-10, an immunosuppressive cytokine following treatment with TCDD when compared to the vehicle controls (**Figure 1E**). In these cultures, we also measured IL-22 because it has been shown to ameliorate liver injury in other acute models (23). These data suggested that TCDD attenuates ConA-induced hepatitis by suppressing inflammatory cytokines and promoting anti-inflammatory cytokines.

### TCDD Treatment Alters the Ratio of Th17/Treg Cells in the Liver

**Figure 2:**
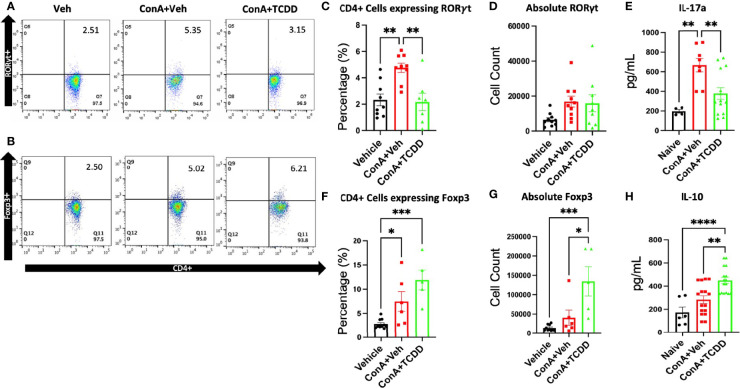
TCDD treatment alters the ratio of Th17/Treg cells in the liver. C57/BL6 mice were injected with ConA followed by vehicle or TCDD 1 hour later as described in **Figure 1** legend. After 24 hours, liver mononuclear cells were harvested and stained for Th17 (CD4+RORγt+) and Treg cells (CD4+Foxp3+). **(A, B)** Representative flow plots showing cells double-stained for the markers. **(C)** Percentage of Th17 cells. **(D)** The absolute number of Th17 cells found per animal. **(E)** Measurement of IL-17A by ELISA in cell cultures. **(F)** Percentage of cells expressing CD4+FoxP3+. **(G)** Total number of cells expressing Foxp3/per mouse. **(H)** Measurement of IL-10 in cultures. (n = 5-10 mice) Significance was determined by one-way ANOVA followed by Tukey’s *post-hoc* test. Vertical bars represent Mean +/- SEM. *p < 0.05, **p < 0.01, ***p < 0.001, ****p < 0.0001.

To assess how TCDD affects the progression of hepatitis, phenotypic changes in the CD4+ lymphocyte subsets were examined specifically regarding the Th17 and Treg balance. Mouse livers were perfused and processed for liver mononuclear cells as described in Methods and were stained and the cells were analyzed by flow cytometry. Live cells that were CD45+CD3+CD4+Foxp3+ were considered to be Tregs and those that were CD45+CD3+CD4+RORγt+ were characterized as Th17. ConA treatment caused a significant increase in the percentage but not the absolute numbers of CD45+CD3+CD4+ RORγt+ Th17 cells, while TCDD treatment caused a significant decrease in the percentage of such cells (**Figures 2A, C, D**). Further, TCDD significantly increased the percentage (**Figures 2B, F**) as well as the absolute number (**Figure 2G**) of Foxp3+ Tregs when compared to the controls.

Next, we measured the production of cytokines, IL-17A and IL-10, by the liver mononuclear cells isolated from mice in each treatment group cultured *in vitro*. The culture supernatants tested using ELISA showed that TCDD treated mice had increased secretion of IL-10 (**Figure 2H**), as well as a decrease in the concentration of IL-17A, the signature cytokine of Th17 cells (**Figure 2E**) when compared to the controls. Together, these data demonstrated that TCDD increases the percentage and numbers of Tregs while decreasing the percentages of Th17 cells.

### ScRNA-Seq Reveals That ConA Alters T Cell and B Cell Phenotypes

**Figure 3:**
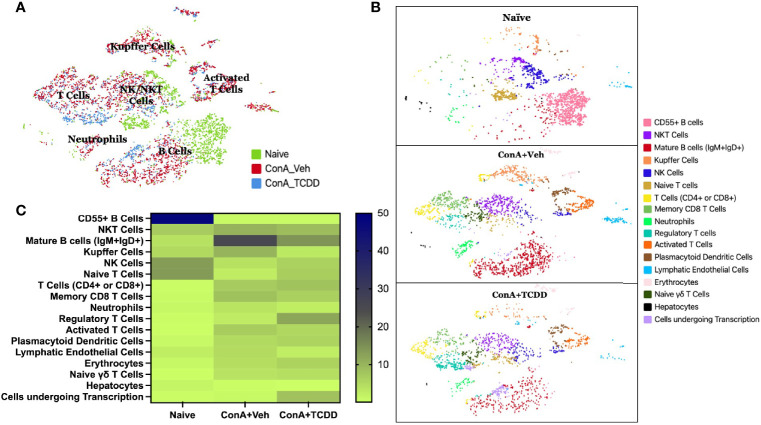
Identification of changes in cellular types following ConA-induced hepatitis using scRNA-seq. C57/BL6 mice were injected with ConA followed by vehicle or TCDD 1 hour later as described in **Figure 1** legend. Liver infiltrating cells were isolated and scRNA-seq was performed as described in Methods. **(A)** tSNE plot with each treatment group separated by color and main clusters labeled. **(B)** tSNE plot separated by sample group and colored to show varying stages of activation and differentiation **(C)** Heatmap of the percentages of cells contained in each tSNE cluster with the increases in the percentage shown by the change in color from light to dark.

scRNA-sequencing was performed on isolated liver infiltrating mononuclear cells to further examine the phenotypic immunological changes observed upon ConA challenge and TCDD treatment. A total of 2086 cells from the Naïve sample, 2779 cells from the ConA+Veh sample, and 1880 cells from the ConA+TCDD sample were captured and sequenced. The t-distributed stochastic neighbor embedding (tSNE) statistical method was used to generate the plot annotating each sample by color (**Figure 3A**). We observed that ConA-treated mice showed unique clusters of immune cells when compared to the naïve mice and there were significant differences noted following TCDD treatment (**Figure 3B**). For example, we observed CD55+ B cells in the naïve mice which were completely lacking in ConA+vehicle and ConA+TCDD treated groups (**Figure 3B**). Also, the ConA+vehicle group had mature B cells (IgM+IgD+) which were deficient in the Naïve group and were markedly decreased in the ConA+TCDD group (**Figure 3B**). Additionally, NKT cells were predominantly present in the ConA+vehicle group when compared to the Naïve group while the ConA+TCDD group showed a significant decrease in this population (**Figure 3B**).

**Figure 3C** shows data following normalization of the percentage of cells per cluster using the total number of captured events. These data demonstrated a depletion of CD55+ B cells upon ConA challenge when compared to the naïve mice and ConA+TCDD group (**Figure 3C**). Specifically, 29.96% of the cells observed in the Naïve group were CD55+ B cells, while those in the ConA+Vehicle and ConA+TCDD group made up 0.07% and 0.21% respectively (**Figure 3C**). There was also a higher percentage of NKT cells in the ConA+Veh group as compared to the others (**Table 1**). Consistent with our flow cytometry data, we observed an increase in the percentage of Tregs upon TCDD treatment (Naïve: 0.48%, ConA+Veh: 2.70%, ConA+TCDD: 11.76%). Together, these data suggested that ConA, a polyclonal activator of T cells, can also alter the presence of various types of immune cells in the liver, and furthermore, TCDD can reverse some of these changes.

**Table 1:** Percentage of cells in each cluster from scRNA-seq.

Column1	Naïve	ConA+Veh	ConA+TCDD
CD55+ B Cells	47.84%	0.68%	0.74%
NKT Cells	7.00%	10.11%	8.78%
Mature B cells (IgM+IgD+)	3.88%	25.08%	14.36%
Kupffer Cells	5.13%	10.08%	3.03%
NK Cells	13.18%	4.07%	6.22%
Naive T Cells	13.37%	2.52%	6.60%
T Cells (CD4+ or CD8+)	0.48%	6.94%	8.09%
Memory CD8 T Cells	0.77%	8.13%	6.28%
Neutrophils	1.10%	3.67%	2.29%
Regulatory T Cells	0.48%	2.70%	11.76%
Activated T Cells	0.19%	6.77%	5.21%
Plasmacytoid Dendritic Cells	1.01%	5.04%	5.74%
Lymphatic Endothelial Cells	2.35%	4.82%	2.39%
Erythrocytes	1.10%	4.39%	5.85%
Naive γδ T cells	0.29%	3.74%	4.31%
Hepatocytes	1.44%	0.40%	0.11%
Cells undergoing Transcription	0.38%	0.86%	8.24%

### Fcer1g, Ptma, Hspe1, and Tcea1 as Significantly Dysregulated Genes in NKT, Mature B Cells, and CD4/CD8 T Cell Clusters

**Figure 4:**
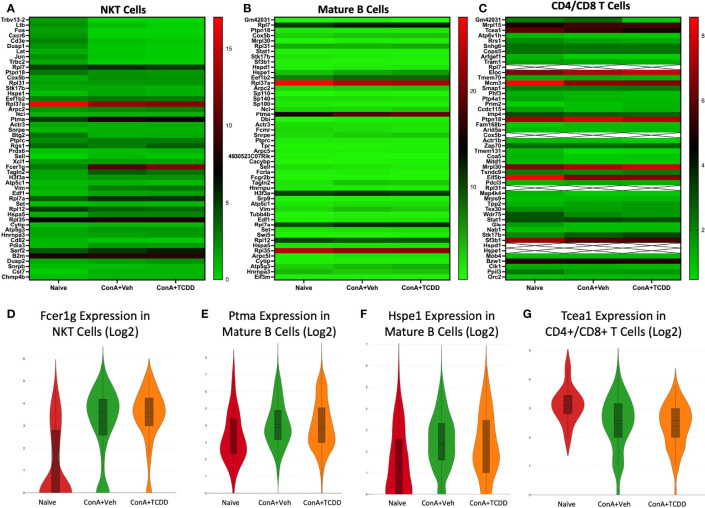
Analysis of changes in gene expression in NKT, Mature B cells, and CD4/CD8 T cell clusters. C57/BL6 mice were injected with ConA followed by vehicle or TCDD 1 hour later as described in **Figure 1** legend. Liver infiltrating cells were isolated and scRNA-seq was performed as described in Methods. Heatmaps consisting of the top 50 differentially expressed genes per treatment were generated using Loupe Browser. Heatmaps of differentially expressed genes from **(A)** NKT, **(B)** Mature B, and **(C)** CD4/CD8 T cells. In panel **(C)**, the X marked with some genes represents an expression level over 20. **(D-G)** Violin plot of Log2 *expression* of target genes.

We created heatmaps consisting of the top 50 differentially expressed genes per treatment group for each cluster. The known functions of the significantly altered genes has been shown in **Table 2**. Within the NKT cell cluster, the expression of *Fcer1g*, a regulatory gene associated with CD8+ T cell function (24), was upregulated with ConA challenge and further upregulated upon TCDD treatment (**Figures 4A, D**). Within the mature B cells cluster, prothymosin-α (*Ptma)* and heat shock protein family E member 1 (*Hspe1)* were identified as genes of interest due to significant changes observed in their expression levels per group (**Figure 4B**). *Ptma*, a gene considered to have immunostimulant properties specifically in terms of IL-2 expression and induction of Th1 responses (25), was upregulated upon ConA exposure in Mature B cells when compared to the naïve group (ConA+Veh mean: 4.06, Naïve mean: 3.3) and downregulated upon TCDD treatment (ConA+TCDD mean: 3.98) (**Figure 4E**). Also, *Hspe1*, shown to be involved in apoptosis and suppression of T cell activation (26), was upregulated in the ConA+Veh group when compared to the Naïve group and was downregulated ConA+TCDD group (**Figure 4F**). In the cluster containing CD4+ and CD8+ T cells, we observed downregulation of transcription elongation factor A1 *(Tcea1)* expression in the ConA+Veh group when compared to the Naïve group and was not altered further in the ConA+TCDD group (**Figures 4C, G**). This gene has been implicated in myeloid cells proliferation, differentiation, and survival (27), though its role in T cells is unknown.

**Table 2:** Target genes and functions.

Gene	Associated Function
*Fcer1g*	Limit CD8+ T cell expansion^24^
*Ptma*	Induce Th1 response^25^
*Hspe1*	Suppress T cell activation^26^ Involved in apoptosis^86^ Induce Tregs^87^
*Tcea1*	Proliferation, differentiation, and survival of myeloid cells^27^
*Lyz2*	Bacteriolytic enzyme found in macrophages^88^
*C1qb*	Drive macrophage polarization^28,89^
*Srgn*	Regulate secretion of inflammatory mediators and promotes tumor aggression^29,30,90^
*Gzma*	Induce apoptosis/cytolysis^31^
*Tmsb4x*	Cellular migration and repair^32,91^
*Ccl5*	Recruit hepatic leukocytes^92^
*Cd52*	Inhibit activation of NF-κB^75^
*Slpi*	Inhibition of neutrophil infiltration^34^
*Il1b*	Inflammatory cytokine^93^
*Cxcr2*	Neutrophil migration^35^
*Rbm3*	Induce apoptosis^36^
*Aldoa*	Infiltration of leukocytes^37^
*Mt1*	Alter the Th17/Treg balance^38^
*Rgs1*	Influence migration and autoimmunity^39^
*Icos*	T cell activation^40^
*Cd28*	T cell co-stimulatory molecule^94^
*Ctla4*	Immune cell infiltration and checkpoint^41^
*Cd37*	Dendritic cell migration^42^
*Actb*	Cytoskeleton housekeeping protein associated with cancer metastasis^95^
*Cxcl10*	Promote T cell-endothelial cell adhesion^43^
*Rpl37a*	Suppress tumor cell proliferation via p53 activation^96^

### Gene Expression Changes Observed in Kupffer Cells and NK Cells Upon ConA Exposure and Subsequent TCDD Treatment

**Figure 5:**
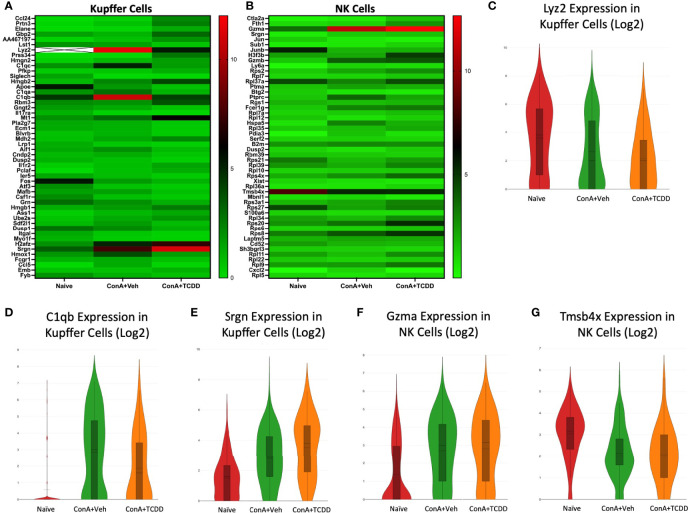
Gene expression changes observed in Kupffer cells and NK cells upon ConA exposure and subsequent TCDD treatment. C57/BL6 mice were injected with ConA followed by vehicle or TCDD 1 hour later as described in **Figure 1** legend. Liver mononuclear cells were isolated and scRNA-seq was performed as described in Methods. Heatmaps consisting of the top 50 differential genes per treatment were generated using Loupe Browser. Heatmaps of differentially expressed genes from **(A)** Kupffer cells and **(B)** NK cells. In panel **(A)**, the X marked with some genes represents an expression level over 20. **(C–G)** Violin plot of Log2 expression of target genes.

Within the Kupffer cell cluster, lysozyme 2 (*Lyz2)*, complement component 1q B chain (*C1qb)*, and serglycin *(Srgn)* were genes of interest found within the list of the top 50 differential genes (**Figure 5A**). *Lyz2* was downregulated in the ConA+Veh group when compared to Naïve and it was further downregulated upon TCDD exposure (**Figure 5C**). *C1qb*, a known component of the complement shown to be positively correlated with macrophages and CD8+ cells in osteosarcoma (28), was significantly upregulated in the ConA+Veh group when compared to the Naïve and was downregulated with TCDD treatment (**Figure 5D**). Notably, expression of the proteoglycan *Srgn* in the Kupffer cell cluster was markedly upregulated in the ConA+TCDD group (**Figure 5E**), when compared to the other two groups, emphasizing its ability to regulate protease, chemokine, and cytokine secretion (29, 30).

We identified granzyme a *(Gzma)* and thymosin beta-4x *(Tmsb4x)* as differential genes of interest in the NK cell cluster (**Figure 5B**). *Gzma* expression was significantly upregulated upon ConA+Veh exposure (ConA+Veh mean: 2.69 as compared to Naïve mean: 1.36) and further upregulated upon TCDD administration (ConA+TCDD mean: 2.81) (**Figure 5F**). Upregulation of this gene supports studies that suggest that secretion of *Gzma* by NK cells serves as a mechanism for target cell pyroptosis (31). Conversely, *Tmsb4x* expression was decreased in the ConA+Veh group when compared to Naïve and was increased following TCDD exposure (**Figure 5G**). This gene has been shown to promote cell migration but may also play a role in the regeneration and repair of damaged cells (32).

### Srgn, Ccl5, and Cd52 Identified as Dysregulated Genes of Interest in Naïve T Cell and Memory CD8+ Cell Clusters

**Figure 6:**
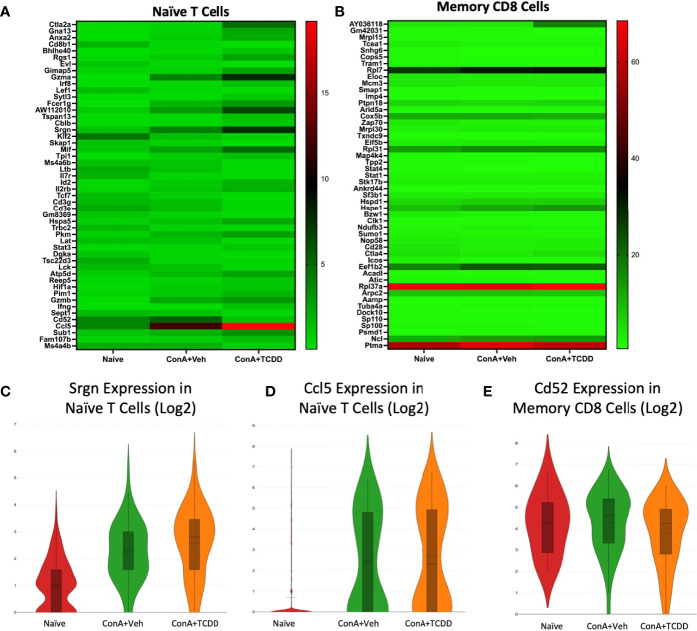
Srgn, Ccl5, and Cd52 identified as dysregulated genes of interest in Naïve T Cell and Memory CD8+ T Cell clusters. C57/BL6 mice were injected with ConA followed by vehicle or TCDD 1 hour later as described in **Figure 1** legend. Liver infiltrating cells were isolated and scRNA-seq was performed as described in Methods. Heatmaps consisting of the top 50 differential genes per treatment were generated using Loupe Browser. Heatmaps of differentially expressed genes from **(A)** Naïve T cells and **(B)** Memory CD8+ T cells. **(C–E)** Violin plot of Log2 expression of target genes.

Our Naïve T cell cluster showed few drastic differences in gene expression (**Figure 6A**). We identified *Srgn* and *Ccl5* as genes of interest. *Srgn* was again upregulated in both ConA exposed groups within this cluster (**Figure 6C**). As expected, *Ccl5* expression was upregulated in Naïve T cells in groups exposed to ConA (**Figure 6D**), supporting the recruitment of leukocytes to the inflamed liver upon activation. In the cluster containing Memory CD8+ T cells, we noticed changes within the expression of *Cd52* (**Figure 6B**). *Cd52* was upregulated in the ConA alone group and downregulated in the ConA+TCDD group (**Figure 6E**). Antibodies against CD52 have been used to decrease the level of surface antigen and treat multiple sclerosis and its murine model experimental autoimmune encephalomyelitis (EAE) (33).

### Analysis of Gene Dysregulation in Neutrophils

**Figure 7:**
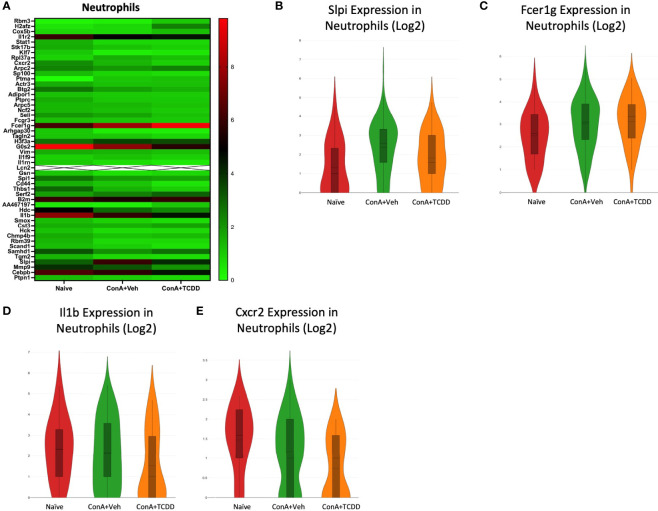
Analysis of gene dysregulation in Neutrophils. C57/BL6 mice were injected with ConA followed by vehicle or TCDD 1 hour later as described in **Figure 1** legend. Liver infiltrating cells were isolated and scRNA-seq was performed as described in Methods. Heatmaps consisting of the top 50 differential genes per treatment were generated using Loupe Browser. Heatmaps of differentially expressed genes from **(A)** Neutrophils. In panel **(A)**, the X marked with some genes represents an expression level over 20. **(B–E)** Violin plot of Log2 expression of target genes.

We observed the transcription profile of the cluster from our scRNA-seq data containing neutrophils. Secretory leukocyte protease inhibitor *(Slpi)*, *Fcer1g*, *Il1b*, and *Cxcr2* were identified as genes of interest due to their considerably different expression levels within each group (**Figure 7A**). *Slpi*, a protease inhibitor that has been shown to inhibit neutrophil infiltration but upregulated in carcinomas (34), was upregulated with ConA+Veh treatment when compared to the Naïve controls and reversed following TCDD treatment (**Figure 7B**). We also observed an increase in *Fcer1g* expression upon ConA exposure when compared to the Naïve control, which was not further altered following TCDD treatment (**Figure 7C**). Interestingly, *Il1b* expression was significantly downregulated in ConA+TCDD treatment when compared to the ConA+Veh group (**Figure 7D**), supporting the ability of TCDD to reduce pro-inflammatory cytokine secretion. Similarly, the cell migratory marker *Cxcr2* was also found to be downregulated in these cells following treatment with TCDD (**Figure 7E**), evidencing the ability of TCDD to reduce migration of neutrophils (35).

### Elucidation of Regulatory T Cell Transcription Profiles

**Figure 8:**
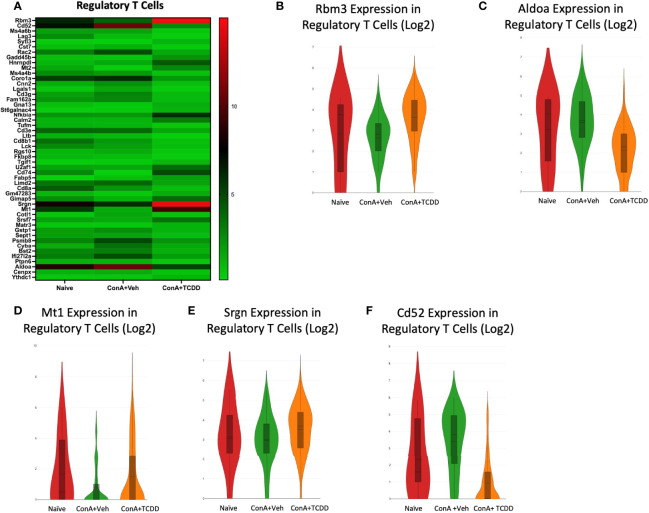
Elucidation of Regulatory T cell transcription profiles. C57/BL6 mice were injected with ConA followed by vehicle or TCDD 1 hour later as described in **Figure 1** legend. Liver infiltrating cells were isolated and scRNA-seq was performed as described in Methods. Heatmaps consisting of the top 50 differential genes per treatment were generated using Loupe Browser. Heatmaps of differentially expressed genes from **(A)** Regulatory T cells. **(B–F)** Violin plot of Log2 expression of target genes.

Upon observing the top 50 differential genes in the Regulatory T cell group, we selected RNA-binding motif 3 *(Rbm3)*, aldolase a (*Aldoa)*, metallothionein 1 (*Mt1)*, *Srgn*, and *Cd52* as genes of interest (**Figure 8A**). *Rbm3*, a gene that has been implicated in apoptosis (36), was downregulated with ConA exposure when compared to Naïve and was upregulated upon TCDD treatment (**Figure 8B**). *Aldoa* was downregulated in the ConA+TCDD group when compared to ConA+Veh (**Figure 8C**), supporting that TCDD plays a role in reducing infiltration of immune cells since the induction of this marker is associated with this mechanism (37). *MT1*, a gene shown to play a role in the suppression of rheumatoid arthritis (38), was downregulated in the ConA+Veh group and upregulated upon TCDD treatment (**Figure 8D**). *Srgn* expression was again upregulated upon TCDD treatment in regulatory T cells when compared to ConA+Veh (**Figure 8E**). As shown in other subsets, *Cd52* expression was downregulated in the ConA+TCDD group when compared to the ConA+Veh group (**Figure 8F**).

### Rgs1, Icos, Cd28, and Ctla4 in Activated T Cells and Cd37 and Actb Expression in Plasmacytoid Dendritic Cells

**Figure 9:**
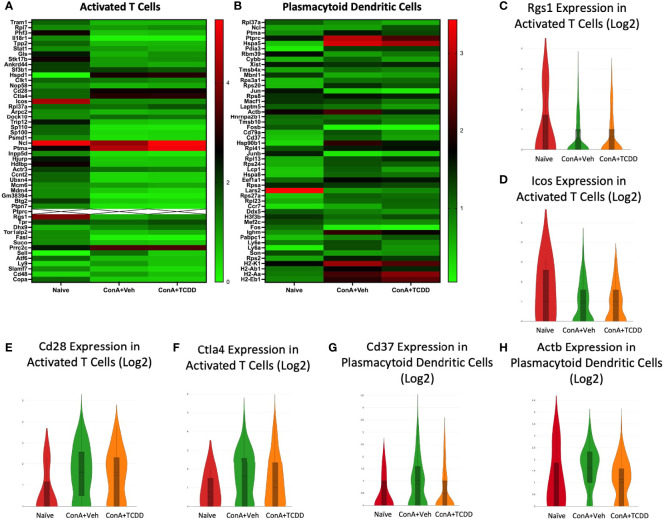
Rgs1, Icos, Cd28, and Ctla4 in Activated T cells and Cd37 and Actb expression in Plasmacytoid Dendritic Cells. C57/BL6 mice were injected with ConA followed by vehicle or TCDD 1 hour later as described in **Figure 1** legend. Liver infiltrating cells were isolated and scRNA-seq was performed as described in Methods. Heatmaps consisting of the top 50 differentially expressed genes per treatment were generated using Loupe Browser. Heatmaps of differentially expressed genes from **(A)** Activated T cells and **(B)** Plasmacytoid Dendritic Cells. In panel **(A)**, the X marked with some genes represents an expression level over 9. **(C–H)** Violin plot of Log2 expression of target genes.

We selected regulator of G-protein signaling 1 (*Rgs1*), inducible T-cell costimulator (*Icos*), Cd28, and cytotoxic T-lymphocyte-associated protein 4 (*Ctla4*) from the heatmaps created from the Activated T cell cluster (**Figure 9A**). Both *Rgs1*, a gene that has been shown to influence migration, reduce the frequency of T follicular helper cells upon knockdown, and play a role in autoimmunity (39), and *Icos*, whose involvement in T cell activation was discovered in 1999 (40), were downregulated in the groups exposed to ConA+Veh when compared to Naïve while TCDD did not further alter their expression (**Figures 9C, D**). The costimulatory molecules *Cd28* and *Ctla4*, immune cell infiltration and checkpoint molecules (41), were upregulated upon ConA challenge but were reduced in the TCDD-treated group (**Figures 9E, F**). Together, these data supported the ability of TCDD to reduce immune cell infiltration and migration.

In the cluster containing plasmacytoid dendritic cells (pDC), we noted a decrease in *Cd37* expression upon TCDD treatment (**Figure 9G**) as well as a decrease in expression of *Actb* when compared to the ConA+Veh (**Figures 9B, H**). CD37 has been shown to control dendritic cell migration (42), again emphasizing TCDD’s role in affecting migration.

### Transcription Profiles of Lymphatic Endothelial Cells and Naïve Gamma Delta T Cells

**Figure 10:**
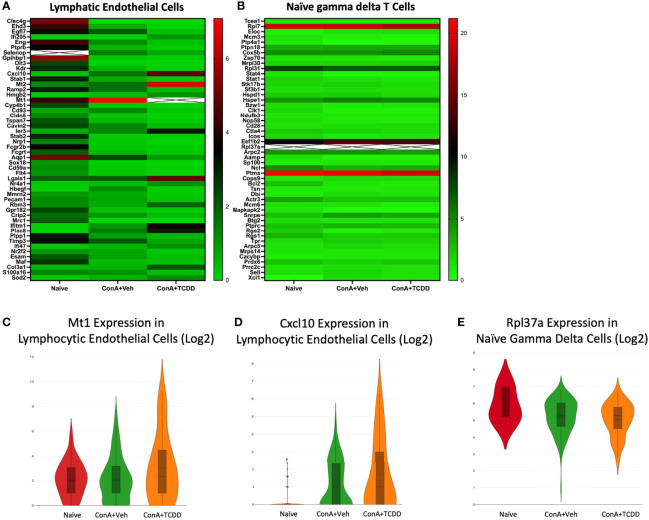
Transcription Profiles of Lymphatic Endothelial Cells and Naïve Gamma Delta T Cells. C57/BL6 mice were injected with ConA followed by vehicle or TCDD 1 hour later as described in **Figure 1** legend. Liver infiltrating cells were isolated and scRNA-seq was performed as described in Methods. Heatmaps consisting of the top 50 differentially expressed genes per treatment were generated using Loupe Browser. Heatmaps of differentially expressed genes from **(A)** Lymphatic Endothelial Cells and **(B)** Naïve Gamma Delta T cells. In panels **(A, B)**, the X marked with some genes represents an expression level over 13. **(C-E)** Violin plot of Log2 expression of target genes.

The profiles of the lymphatic endothelial cells (**Figure 10A**) and naïve gamma delta T cell (**Figure 10B**) clusters were analyzed, and gene targets were selected. Within the lymphatic endothelial cells, we noticed an increase in the expression of *MT1* upon the ConA+Veh challenge that was further upregulated with ConA+TCDD treatment (**Figure 10C**). The upregulation of *MT1* in the ConA alone group is contrary to our observations in the cluster containing Regulatory T cells. We also observed a drastic induction of *Cxcl10* expression upon ConA+Veh exposure when compared to Naïve controls that was further increased in the ConA+TCDD-treated group (**Figure 10D**). CXCL10 is chemokine shown to promote T cell adhesion to endothelial cells and when expressed by Kupffer cells, contributes to ConA-induced hepatitis (43). However, its role is unclear when expressed by endothelial cells.

In several of our clusters, we noticed a very high expression of ribosomal protein L37a (*Rpl37a)* (this gene has a corresponding ‘X’ in **Figure 10B** due to very high levels of expression). We noticed this also in the expression of *Rpl37a* in the naïve gamma delta T cell cluster (**Figure 10B**). Specifically, expression was the highest in the naïve group (46.9) and was downregulated in the ConA+Veh exposed groups to 37.9, and in the ConA+TCDD groups to 39.5 (**Figure 10E**). In breast cancer, high Rpl37a expression has been associated with response to treatment and good prognosis and has been suggested as a biomarker (44, 45) while its role in inflammation remains unclear.

### Trajectory Analysis of NK and T Cell Subsets Uncovers Reduced Proliferation of CD8+ T Cells With TCDD Treatment

**Figure 11:**
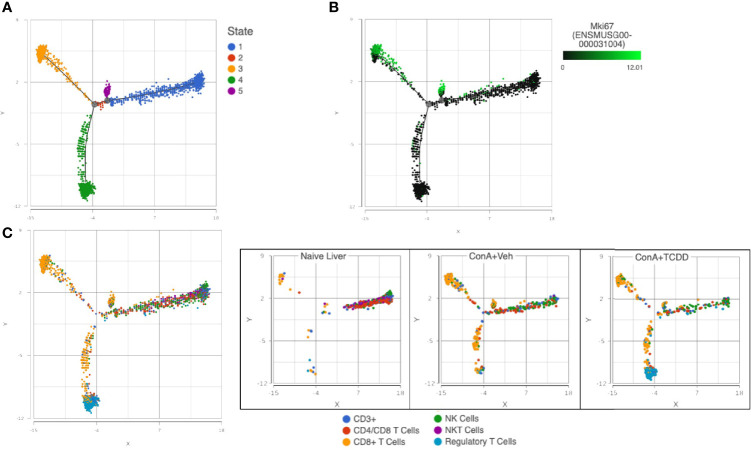
Trajectory Analysis of NK and T cell subsets uncovers reduced proliferation of CD8+ T cells with TCDD treatment. C57/BL6 mice were injected with ConA followed by vehicle or TCDD 1 hour later as described in **Figure 1** legend. Liver infiltrating cells were isolated and scRNA-seq was performed as described in Methods. Monocle 2 within *Partek^®^ Flow^®^* software v10.0 was used for trajectory analysis. **(A)** Pseudotime plots determine 5 different states for T cells and NK cells. **(B)** States 3 and 5 were shown to contain the highest expression of *Mki67*, a gene that encodes for KI-67, a marker of proliferation. **(C)** Colored by cluster, states 3 and 5 contained predominantly CD8+ T cells and TCDD was shown to decrease the amount of these actively proliferating cells.

Using the files generated from our single-cell RNA-sequencing, we used *Partek^®^ Flow^®^* software v10.0 to perform group-based trajectory analysis on NK and T cell subsets (46). Using Monacle 2, five different states were observed (**Figure 11A**). Using this plot, we assessed the expression of *Mki67* to determine cells actively proliferating and found that these cells were observed in states 3 and 5 (**Figure 11B**). We then identified CD8+ T cells as the predominant cell type in these groups and observed an increase in this subset upon ConA+Veh exposure that was decreased in the ConA+TCDD-treated group (**Figure 11C**). These results showed that TCDD is capable of decreasing the amount of actively proliferating CD8+ cells in ConA-induced T cell-mediated liver injury.

## Discussion

ConA-induced liver injury is a murine model that properly replicates human autoimmune hepatitis (47). ConA is a polyclonal activator of T cells that causes massive cytokine release and the recruitment of additional immune cells such as the NKT cell resulting in liver injury (48). AhR agonism suppresses inflammation through a variety of mechanisms, including through inducing Tregs, driving immune cell apoptosis, and suppressing production of pro-inflammatory cytokines (9). TCDD, a strong AhR agonist, has been shown to reduce the secretion of proinflammatory cytokines and shift the Th17/Treg balance toward the immunotolerant Treg phenotype in a variety of inflammatory disease models (8, 49, 50). For these reasons, we explored the effect of AhR activation by TCDD in this model of liver injury.

In this study, we report that TCDD attenuates ConA-induced murine hepatitis *via* the promotion of immunoregulatory processes within the liver. Symptomology of AIH was shown to be decreased in TCDD treated mice, as measured by a decrease in ALT levels, decrease in cell infiltration, and decrease in the production of inflammatory cytokines such as IL-6 and IL-17, antiparallel to an increase in immunosuppressive cytokines such as IL-10. We also observed that while ConA activation caused significant upregulation of IL-22, TCDD treatment caused a further increase in IL-22. IL-22 is a member of the IL-10 cytokine family and is produced by lymphocytes, including activated T cells (51). It enacts its effects on epithelial cells and has been shown to activate the JAK-STAT pathway (52) in addition to inhibiting apoptosis and modulating metabolic function (53). In the liver, it is well established that IL-22 plays a role in liver disease either in a protective manner, through regulation of genes involved in tissue repair, metabolism, and inflammation (54, 55), or in exacerbation of disease as evidenced by upregulation in patient livers of those with chronic hepatitis B and C as well as in hepatitis B virus transgenic mice (56, 57). Interestingly, gene delivery of IL-22 has been shown to prevent liver damage induced by ConA (58). Based on this, our data suggest that the TCDD-mediated increase in IL-22 may play a role in preventing liver injury caused by ConA.

In an earlier study, TCDD was found to exacerbate ConA-induced liver injury (59). The reason for this discrepancy may stem from the fact that in this study, the authors pretreated normal mice with TCDD, and 4-10 days later, injected ConA. Also, they used 0.3, 3, or 30 μg/kg TCDD and 6mg/kg Con A. TCDD injection in normal mice has been shown to alter the immune response significantly. For example, TCDD causes thymic atrophy by day 5 when injected into normal mice (12, 13, 60–62). Also, Foxp3^+^ Treg cells primarily develop in the thymus, and the thymic Treg cell pool is composed of not only newly developing Tregs but also recirculating peripheral cells (63). Thus, pretreatment with TCDD may deplete Tregs leading to increased hepatitis, a suggestion that remains to be validated.

In another study, Nault et al. performed single-nuclei RNA sequencing on mice treated with TCDD every 4 days for 28 days at a dose of 30 μg/kg (64). This study reported an increase in immune cell infiltration upon TCDD treatment whereas our study showed a reduction. These contrasting findings may be due to the long-term exposure to TCDD at a higher dose in naïve animals as compared to our 24-hour, single dose model in mice exposed to ConA, a polyclonal activator of T cells. Previous studies have shown that naïve *vs* activated T cells show differential susceptibility to TCDD-mediated toxicity (12). Further, it is known that long-term TCDD exposure results in different pharmacokinetics in the liver as compared to acute exposure (65).

In order to determine the underlying processes by which TCDD suppresses symptomology in a murine model of autoimmune liver disease, we applied scRNA-seq to identify the cellular and molecular mechanisms by which TCDD mediates liver-infiltrating leukocyte function. In the current study, the use of scRNA-seq led to some surprising observations such as the changes occurring in the B cell compartment, especially CD55+ B cells, because ConA is a polyclonal T cell mitogen. CD55, also called Decay Accelerating Factor, is a regulatory protein involved in complement activation and has been shown to be modulated in many infections and diseases (66–69). An increase in the surface expression of CD55 has been associated with inhibition of complement system activation (69). It has been reported that a decrease in the mean fluorescence intensity of CD55 was observed in T and B lymphocytes from systemic lupus erythematosus patients, implicating this marker as a possible player in lymphopenia (70). The depletion of CD55+ B cells in mice exposed to ConA and an inability of TCDD to restore this population suggest a possible role of complement activation in disease pathogenesis that future studies should aim to address.

Although studies have associated TCDD with amelioration of disease (8, 71–73), we demonstrate that treatment of ConA-induced liver injury with TCDD results in distinct gene expression profiles as compared to ConA challenge alone. Specifically, many genes associated with migration and activation of immune cells were found to be altered upon ConA challenge and TCDD treatment. We also found that *Srgn* was the top differentially expressed gene in multiple clusters, possibly implicating a more pronounced role in the pathogenesis of ConA-induced hepatitis. *Ptma* has been shown to inhibit transforming growth factor-beta (TGF-β) signaling (74) and transduction of this gene attenuated inflammation in a rat model of pulmonary hypertension (75). Additionally, evidence supports the critical role of *Hspe1* as an inhibitor of inflammation through suppression of T cell activation (76). Finally, through inhibition of nuclear factor kappa B (NF-κB) activation and induction of apoptosis, *Cd52* has been shown to suppress inflammation (77). Alterations in the expression of these genes by TCDD suggest their involvement in the suppression of inflammation.

We show that TCDD reduces the percentage of NKT, Kupffer, and activated T cells in the present study. In the ConA-induced model of AIH, these cell types are frequently involved in inducing inflammation and injury (78–81). The ability of TCDD to reverse the induction of these cell types caused by the ConA challenge suggests a cellular mechanism through which suppression of inflammation may occur in this model. Further, it is well known that CD8+ T cells play a role in the inflammatory response (82, 83). These cells are involved in effector functions and have been shown to exacerbate inflammation in various models (84–86). Here, we show that these cells are proliferating at a higher level in ConA-induced hepatitis as evidenced by the increase in expression of *Mki67* (**Figure 11A**). The ability of TCDD to reduce these proliferating cells supports studies implicating the involvement of this compound in the suppression of cellular proliferation (87).

While our findings are exciting, there exists some limitations of this study. The ConA-hepatitis model is a well-established model for AIH in mice, however, it is acute, so symptoms disappear after 48 hours and there is no production of autoantibodies (88). Thus, it does not truly mimic AIH in humans in this regard. Another limitation of this study is that while the dose used is relevant to reduce inflammation, it is higher than the physiologically relevant protocol established for mice which is 20 ng/kg TCDD twice weekly (89) derived from the background exposure level of < 10 pg/g TCDD in humans (90). However, the goal of the current study was to address mechanistically how AhR activation leads to attenuation of AIH so that new AhR agonists can be developed to treat AIH.

Collectively, this study suggests that scRNA-seq is a powerful technique to study various immune cell types during disease. While ConA is considered to be a T cell mitogen, it was interesting to see changes occurring in various immune cell types including B cells using scRNA-seq. Also, the scRNA-seq provided important data on changes occurring at the transcriptional level at individual cell types. The use of TCDD, a potent AhR ligand also helped address the mechanisms through which AhR activation leads to attenuation of ConA-induced hepatitis.

## Data Availability Statement

The datasets presented in this study can be found in online repositories. The name of the repository and accession number can be found below: NCBI Gene Expression Omnibus; accession number GSE201006.

## Ethics Statement

The animal study was reviewed and approved by University of South Carolina Institutional Animal Care and Use Committee.

## Author Contributions

Conceptualization: AC, PN, and MN; Experimentation and data acquisition: AC, BH, KW, and KM; Validation: AC; Formal analysis: AC; Resources: PN and MN; Writing- original draft: AC; Writing- review and editing: AC, ND, PN, and MN; Visualization: AC; Supervision: PN and MN; Funding acquisition: PN and MN. All authors contributed to the article and approved the submitted version.

## Funding

This study was funded by National Institutes of Health grants R01ES019313, R01MH094755, R01AI123947, R01AI129788, P01AT003961, P20GM103641, R01AT006888, and 3R01AI123947-04S1 (PN and MN).

## Conflict of Interest

The authors declare that the research was conducted in the absence of any commercial or financial relationships that could be construed as a potential conflict of interest.

## Publisher’s Note

All claims expressed in this article are solely those of the authors and do not necessarily represent those of their affiliated organizations, or those of the publisher, the editors and the reviewers. Any product that may be evaluated in this article, or claim that may be made by its manufacturer, is not guaranteed or endorsed by the publisher.
